# Physiotherapists’ use and perceptions of digital remote physiotherapy during COVID-19 lockdown in Switzerland: an online cross-sectional survey

**DOI:** 10.1186/s40945-021-00112-3

**Published:** 2021-07-07

**Authors:** Anne-Kathrin Rausch, Heiner Baur, Leah Reicherzer, Markus Wirz, Fabienne Keller, Emmanuelle Opsommer, Veronika Schoeb, Stefano Vercelli, Marco Barbero

**Affiliations:** 1 ZHAW Zurich University of Applied Sciences, School of Health Professions, Institute of Physiotherapy, Research & Development, Katharina-Sulzer-Platz 9, CH 8401 Winterthur, Switzerland; 2 Department of Health Professions, Physiotherapy, Bern University of Applied Sciences, Murtenstrasse 10, CH-30008 Bern, Switzerland; 3 School of Health Sciences (HESAV) - University of Applied Sciences and Arts Western Switzerland (HES-SO), Avenue de Beaumont 21, CH-1011 Lausanne, Switzerland; 4 Department of Business Economics, Rehabilitation Research Laboratory 2rLab, Health and Social Care, University of Applied Sciences and Arts of Southern Switzerland, Stabile Piazzetta, via Violino 11, CH-6928 Manno/Landquart, Switzerland

**Keywords:** Pandemic, Physical therapy modalities, SARS-CoV-2, Telerehabilitation

## Abstract

**Background:**

The Swiss containment strategy for the COVID-19 pandemic during the first wave in spring 2020 resulted in a moratorium on non-urgent physiotherapy via regular direct patient contact. Consequently, such physiotherapy sessions declined by 84%. This study investigates the impact of this moratorium on the use of digital remote physiotherapy in Switzerland during this period and the perceptions of its use by Swiss physiotherapists (PTs).

**Methods:**

A cross-sectional online questionnaire was distributed between June and August of 2020 via the Swiss Physiotherapy Association (physioswiss) and various associations of physiotherapy specialists (e.g., sport, pediatric) working in both inpatient and outpatient settings. The questionnaire was designed to capture the demographics of participants and the perceptions of PTs using 33 questions in the following domains: Demography; Attitudes towards digital technology; Private and professional use of digital technology; Use of digital technology during therapy; and, Support requirements. Closed and open-ended questions were included and the frequency of answers was analyzed. Non-parametric inferential statistics were used to identify differences, where appropriate. The Checklist for Reporting Results of Internet E-Surveys (CHERRIES) was adopted.

**Results:**

Participants in the survey were 742 PTs (23.5% male, mean age of 43 years, mean working experience of 18 years) from the German-speaking (75.5%), French-speaking (15.1%), and Italian-speaking (9.4%) regions of Switzerland. The percentage of PTs using digital remote therapy increased from 4.9% prior to the lockdown to 44.6% during the lockdown period. The majority of PTs did not consider that digital remote therapy could complement usual physiotherapy practice and did not plan to continue with digital remote therapy after the pandemic.

**Conclusions:**

During the lockdown, Swiss PTs adopted various low-cost and easily accessible digital technologies. However, several barriers hampered further implementation of this modality. Specific education and training programs need to be provided among PTs, appropriate digital technologies should be introduced, and a correct reimbursement scheme should be developed.

**Trial registration:**

COVIDPhysio Registry of World Physiotherapy, registered 15th June 2020 (https://world.physio/covid-19-information-hub/covid-19-covidphysio-registry).

**Supplementary Information:**

The online version contains supplementary material available at 10.1186/s40945-021-00112-3.

## Background

The digital physiotherapy task force of World Physiotherapy defined digital practice as “Health care services, support, and information provided remotely via digital communication and devices” with the aim “to facilitate effective delivery of physical therapy services by improving access to care and information and managing health care resources” ([1], p4).

Based on the results of recent systematic reviews, digital remote therapy should be considered as an alternative to usual face-to-face treatments. Cottrell et al. [2] reported aggregated results suggesting that real-time telerehabilitation contacts reduce pain and improve physical function in a variety of musculoskeletal conditions. In line with these findings, the feasibility and potential of increasing the quality of life of surgical patients has also been confirmed by van Egmond et al. [3]. Notably, cost-effectiveness elements, in the form of reduced hospitalization or healthcare utilization, have been observed when telehealth is adopted in persons with chronic obstructive pulmonary disease or heart failure [4, 5].

Despite these reported benefits, digital remote therapy has not yet been widely adopted by practicing physiotherapists (PTs) in Switzerland (CH). This could be the consequence of a number of barriers to implementation, e.g., lack of reimbursement for implementation and maintenance of digital physiotherapy [6], limited technological literacy [7], preference for a “hands-on-approach” [8], lack of knowledge of physiotherapy processes and workflow by information system developers, and technical tools that do not address practical needs [6].

During the first lockdown due to the SARS CoV-2 pandemic in spring 2020 in CH, there was a moratorium on health professionals (HPs) carrying out non-urgent medical examinations, treatments, and interventions [9, 10]. Consequently, the number of physiotherapy sessions per week in outpatient practices fell by 84% [11] and a high proportion of PTs submitted requests for an indemnity due to reduced working hours [12]. Swiss PTs, however, were still able to provide and invoice remote therapy to COVID-19 survivors or other patients with an urgent need of continuous therapy [13, 14]. Thus, despite posing many challenges, the pandemic situation also provided Swiss PTs with the opportunity to gain experience in using digital technologies for remote therapy. The digitalization of physiotherapy, which has already been implemented in many other countries [15–17], was now also observed in CH. The aim of this study is to analyze the perceptions of Swiss PTs on the use of digital technologies in the context of their practices during the COVID-19 pandemic of spring 2020 in CH.

## Methods

### Study design

This cross-sectional survey was conducted with PTs practicing in CH. The online software EFS survey from QuestBackUnipark (https://www.unipark.com/, Cologne, Germany) was used for digital and anonymous collection of data [18]. The findings are reported in line with the Checklist for Reporting Results of Internet E-Surveys (CHERRIES) [19].

### Ethics

According to the Federal regulations (Swiss Human Research Act, 2020), because all data was collected anonymously, ethical approval was not required for this study. Nevertheless, a clarification of responsibility was obtained from the Ethics Committee Zurich (BASEC-No.: Req-2020-00783). Furthermore, the study was registered in the COVIDPhysio Registry of World Physiotherapy as work in progress regarding COVID-19 and Physiotherapy [20].

### Recruitment

The largest Swiss Physiotherapy Association (physioswiss), with approximately 10,000 members, and various associations of professional physiotherapy specialists (e.g., the Swiss Association of Orthopedic Musculoskeletal Physiotherapy, SVOMP; the Swiss Sports Physiotherapy Association, Sportfisio; the Swiss Association of Independent Physiotherapists, ASPI; the Swiss Working Group for Rehabilitation Training, SART; the Swiss Association of Physiotherapists specialized in pediatrics, Physio Paediatrica; and, ALUMNI of Master-classes of the Swiss Universities of Applied Sciences) were contacted and requested to distribute the survey link within their organizations (e.g., via newsletter, social media). Physioswiss also sent a reminder to their members after 4 weeks. In addition, physiotherapy institutes with large inpatient and outpatient departments were contacted personally and asked to promote participation in the survey to their staff. The open online survey was designed to avoid repeat participation by automatically blocking IP addresses that had already been used.

### Survey

The survey covered questions related to the specific situation of Swiss PTs. The domains were developed through discussion and consensus within the working group. An English version of the survey served as the basis for the French, Italian and German translations, which are the three main national languages of CH. Participants were asked to choose their preferred language on opening the online survey. The survey, consisting of 33 dichotomous or multiple-choice questions, multiple answer options, and additional free text fields (“other” options), was subdivided between the following sections:

- Demographic information: age, gender, working experience
- Attitude towards technology: usage of digital tools (type, frequency) for personal and professional purposes
- Working situation of PT: activities (setting, work categories, function) and workload before and during the lockdown
- Usage of technical tools during therapy, in terms of video/teletherapy before and during the lockdown (if ‘yes’: which patients, which phase of therapy, setting, tools, data protection, charging, quality of communication, quality of therapy interventions, future use; if ‘no’: reasons)
- Support (requirements for information/training regarding technology-based therapy)

The questionnaire is attached to this manuscript as supplementary file (S1).

Pretests were performed within 2 weeks with four individuals from each target language group (*n* = 12) for linguistic validation. Small adaptations were made to improve linguistic comprehensibility. The order of questions was maintained constant, with no randomization or alternation. Filter questions were implemented to reduce the number of items to be answered (item-display was based on the answers to previous items, e.g., if respondents answered that they did not use technology, then the next question on frequency of use was skipped). The full questionnaire was displayed in 13 to 16 screens, depending on the answers to the filter questions, with a maximum of four items shown on each screen. Participants were able to review and/or modify their answers through a ‘Back’ button.

### Analysis

Data was exported from the Unipark server as Excel files (Microsoft Office 2016). No IP addresses were collected, thus ensuring both data security and the anonymity of participants. In Excel, the raw data were cleaned. Missing data occurrences were indicated in the results.

Answers in free-text were categorized, quantified, and analyzed by frequency count by two authors independently. Descriptive statistics were calculated for the total sample and used to describe the prevalence of the answers related to the following dimensions: i. Attitude towards technology; ii. Working situation of PT, i.e., the impact of the COVID-19 lockdown on the type of activity performed by a PT and workload; iii. The use of digital technologies to provide remote physiotherapy; and, iv. Support required, i.e., the PT’s perception and evaluation of the use of remote physiotherapy. Variables were described through their frequency and corresponding percentage. Missing data were reported for each variable.

Non-parametric statistics (Mann-Witney U test, Kruskall-Wallis H test for ordinal variables and Chi-square for categorical variables) were performed to find differences between demographic variables (age, gender, language, work experience) and: 1) the frequency of use of digital tools for professional purposes; 2) the use of digital remote physiotherapy during the lockdown; 3) the intention to continue offering digital remote physiotherapy sessions; and 4) to find an association between workload, work duties, and the use of digital tools and digital remote physiotherapy before and during the COVID-19 lockdown. Based on the distribution of the sample, age was dichotomized as < 45 and ≥ 45 years. The significance level was set at *p* < 0.05. Data analysis was performed with SPSS Statistics Version 26.0.

## Results

Participants were able to access the survey between 30th June and 31st August of 2020. In total, the survey received 1393 visits, with a click-through rate of 53%, but 452 visits ending on page one (choice of language) and a further 199 visits not being completed. Finally, data from 742 respondents were recorded and analyzed (response rate 53%, with some 7% being physioswiss members). The demographics and characteristics of the respondents (Table 1) showed that the majority of respondents were female and working in the German-speaking part of CH in an outpatient setting. Participants took 12 min on average to complete the survey. Free-text answers are presented in the Supplement (S2).

**Table 1:** Demographics and general characteristics of the physiotherapists (PT) interviewed (*n* = 742)

Characteristics of respondents	Mean (SD) Min; Max	***n*** = 742 (%)
**Age**	43 (±11) 24; 68	
**Working Experience (years working as PT)**	18 (±12) 0;48	
**Language**		
German		560 (75.5%)
French		112 (15.1%)
Italian		70 (9.4%)
**Gender**		
Female		550 (74.1%)
Male		174 (23.5%)
Unknown		5 (0.7%)
Missing		13 (1.7%)
**Patient contact during Lockdown**		
Yes		699 (94.2%)
No		43 (5.8%)
**Work employment during Lockdown**		
Outpatient		548 (73.9%)
Inpatient		59 (8.0%)
Both		92 (12.4%)
Missing		43 (5.8%)
**Changed professional duties during Lockdown**		
Yes - within field of physiotherapy		57 (7.7%)
Yes - outside the field of physiotherapy		56 (7.5%)
No		586 (79.0%)
Missing		43 (5.8%)

### Attitude towards technology

**Fig. 1:**
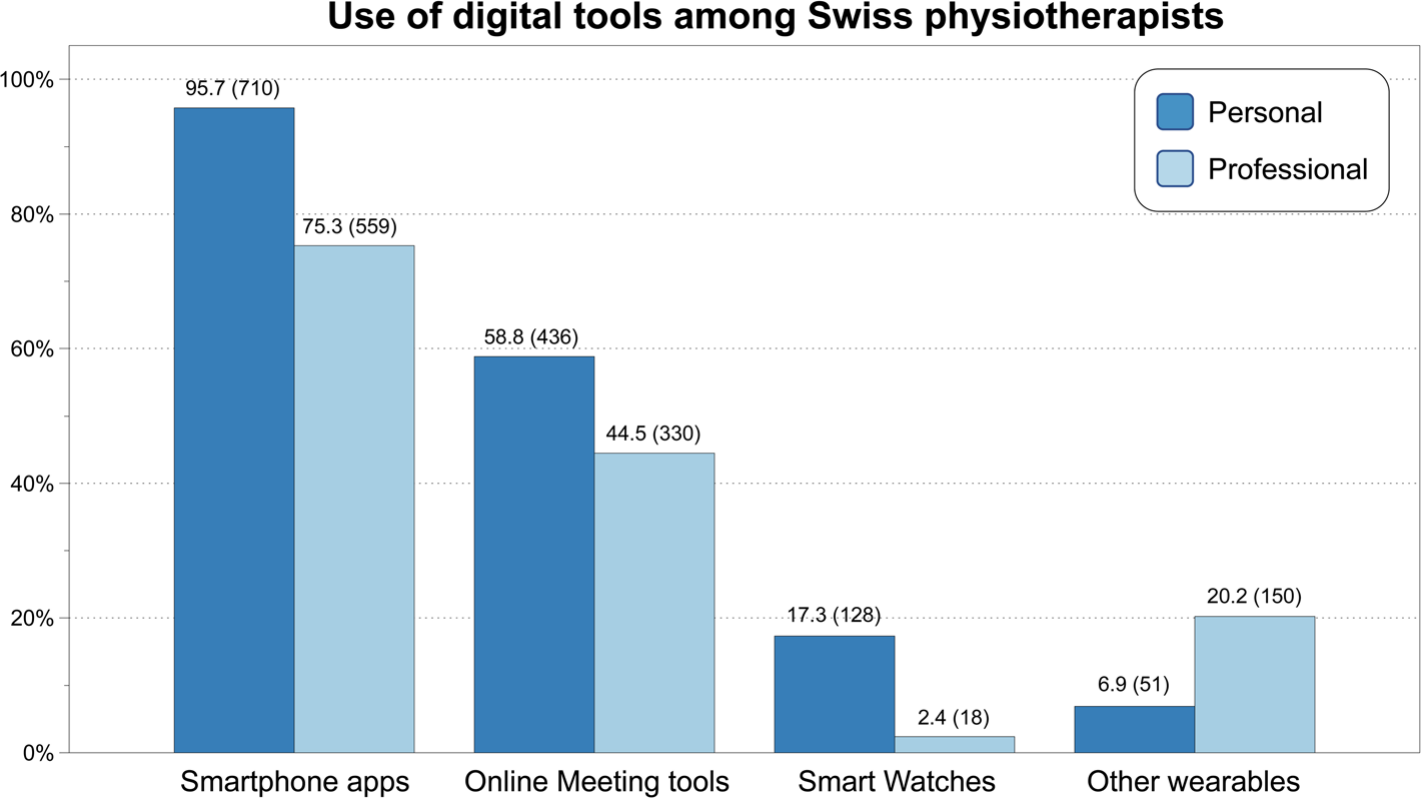
Use of digital tools for personal and professional purposes by Swiss physiotherapists during the spring pandemic (2020); percentage (absolute number)

The use of digital tools by the respondents for personal or professional purposes is summarized in Fig. 1. For personal purposes, almost all respondents stated that they used digital tools daily (92.1%, *n* = 669), or on three to 5 days per week (4.8%, *n* = 35). For professional purposes, 45.3% (*n* = 332) used digital tools daily, 25.8% (*n* = 189) between three to 5 days per week, and 28.9% (*n* = 212) never or less than once per week. Of those respondents using digital tools daily for work, 99% also do so for personal purposes. Participants below 45 years of age (*p* = 0.007) and those with less working experience (*p* = 0.031) used digital tools for professional purposes more often.

#### Working situation of the PT - the impact of COVID-19 lockdown

Prior to the lockdown, 60% (*n* = 448) of PTs worked at least 31 h per week. During the lockdown, there was a statistically significant reduction (x^2^ = 58.511, *p* < 0.001) in workload. During this 10-week period, over 40% of the sample stated that they worked less than 10 h a week, with 21.7% working less than 4 h.

Prior to the lockdown, the PTs’ working activities were 70% clinical, 17.8% administrative, 18.6% research, 14.2% teaching, and 12.9% other tasks. During the lockdown, only 23.6% (*n* = 144) of PTs worked 31 or more hours per week. Activities shifted to 30% clinical work, 26.5% administrative, 23.5% research, 17.9% teaching, and 24.5% other tasks. Of those physiotherapists who changed their working duties within the rehabilitation area (*n* = 57, representing 7.7% of the total sample), only few (*n* = 16, 2.2%) were called to work in a COVID-19 environment.

#### Use of digital technologies to provide remote physiotherapy

Table 2 indicates that the percentage of PTs using remote physiotherapy increased from a prior rate of 4.9% (*n* = 36) to 44.6% (*n* = 332) during the lockdown.

**Table 2:** Frequency analysis of the use of digital technologies to provide remote physiotherapy before and during the lockdown (*n* = 742)

Physiotherapists providing remote care using digital technologies	Counts (n)	Percent (%)	Chi-square	Sig.
**Before lockdown**			29.896	*p* < 0.01
Yes	36	4.9%		
No	663	89.4%		
Missing	43	5.8%		
**During lockdown**				
Yes	332	44.6%		
No	368	49.6%		
Missing	42	5.8%		

A statistically significant correlation was observed between PTs using the remote modality and being aged under 45 (x^2^ = 9.513, *p* = 0.002) and belonging to the German or Italian language groups (x^2^ = 9.628, *p* = 0.008). Remote therapy users (*n* = 332, 44.6%) most frequently delivered the modality in the individual care setting (*n* = 320, 96.4%), compared to group sessions (*n* = 32, 9.6%). The distribution of patient groups receiving remote therapies is reported in Table 3, with the most recipients being COVID-19 high-risk patients and those with musculoskeletal disorders.

**Table 3:** The following proportions are reported: patient groups involved with remote physiotherapy (*n* = 332); forms of support deemed useful by physiotherapists who provided remote therapies during the lockdown (*n* = 332, 44.6%); reasons for not providing remote physiotherapy (*n* = 368, 49.6%)

Patient groups involved with remote physiotherapy	Counts (n)	Percent (%)
Musculoskeletal disorders	226	68.1%
COVID-19 risk group^a^	206	62.0%
Pediatrics	73	22.0%
Geriatrics	63	19.0%
Neuromotor	53	16.0%
Internal organs and vessels	25	7.5%
Patients with COVID-19	22	6.6%
Others	34	10.2%
**Information on digital technology deemed useful by physiotherapists**	**Counts**	**Percent**
Knowledge about infrastructures	289	98.9%
Smartphone applications (apps)	133	45.5%
Law and data protection	214	73.3%
Settlement with cost units (invoice)	225	77.1%
Federal and Cantonal ordinances	218	74.7%
Knowledge about needs of patients	99	33.9%
Knowledge of patient’s requirements (technical)	93	31.8%
Effectiveness of remote therapy	62	21.2%
Communication methods	130	44.5%
Examination and treatment process	105	36.0%
Suitable methods	94	32.2%
Other	143	49.0%
**Reasons for not providing remote physiotherapy**	**Counts**	**Percent**
I was able to provide my patients with sufficient care in another way	99	26.9%
I miss the tactile control/possibility of manual support	63	17.1%
The necessary infrastructure is missing for me or my patients	47	12.8%
Remote physiotherapy is not adequately reimbursed	37	10.1%
I cannot observe the patient adequately	24	6.5%
The technical possibilities are unknown to me or my patients	12	3.3%
Other reasons	86	23.3%

^a^persons over 65 years of age and/or previous illnesses such as chronic respiratory diseases, diabetes, cardiovascular diseases, cancer and/or with a weakened immune system

**Fig. 2:**
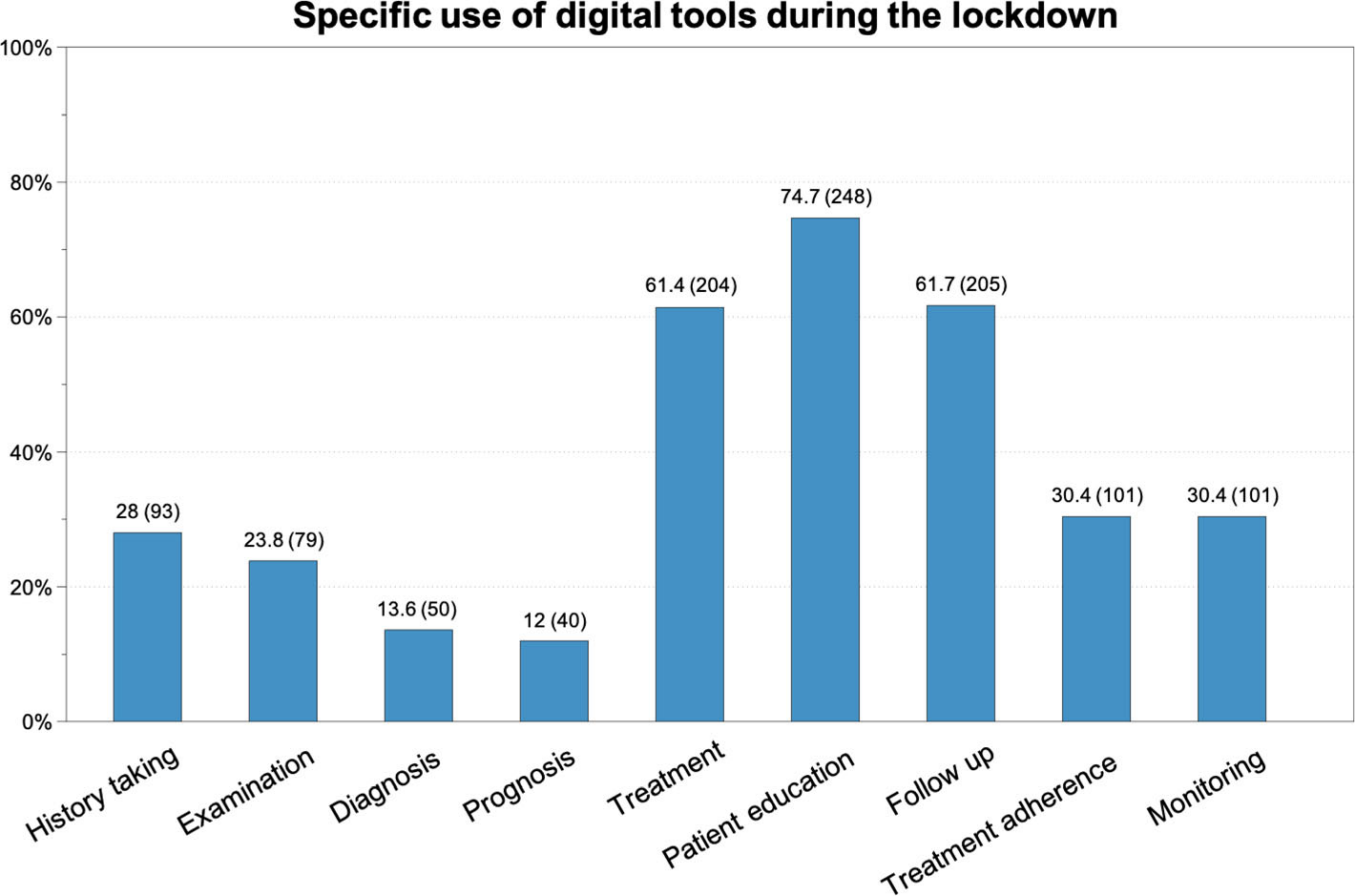
Specific use of digital tools according to the task of treatment during the lockdown; percentage (absolute number)

**Fig. 3:**
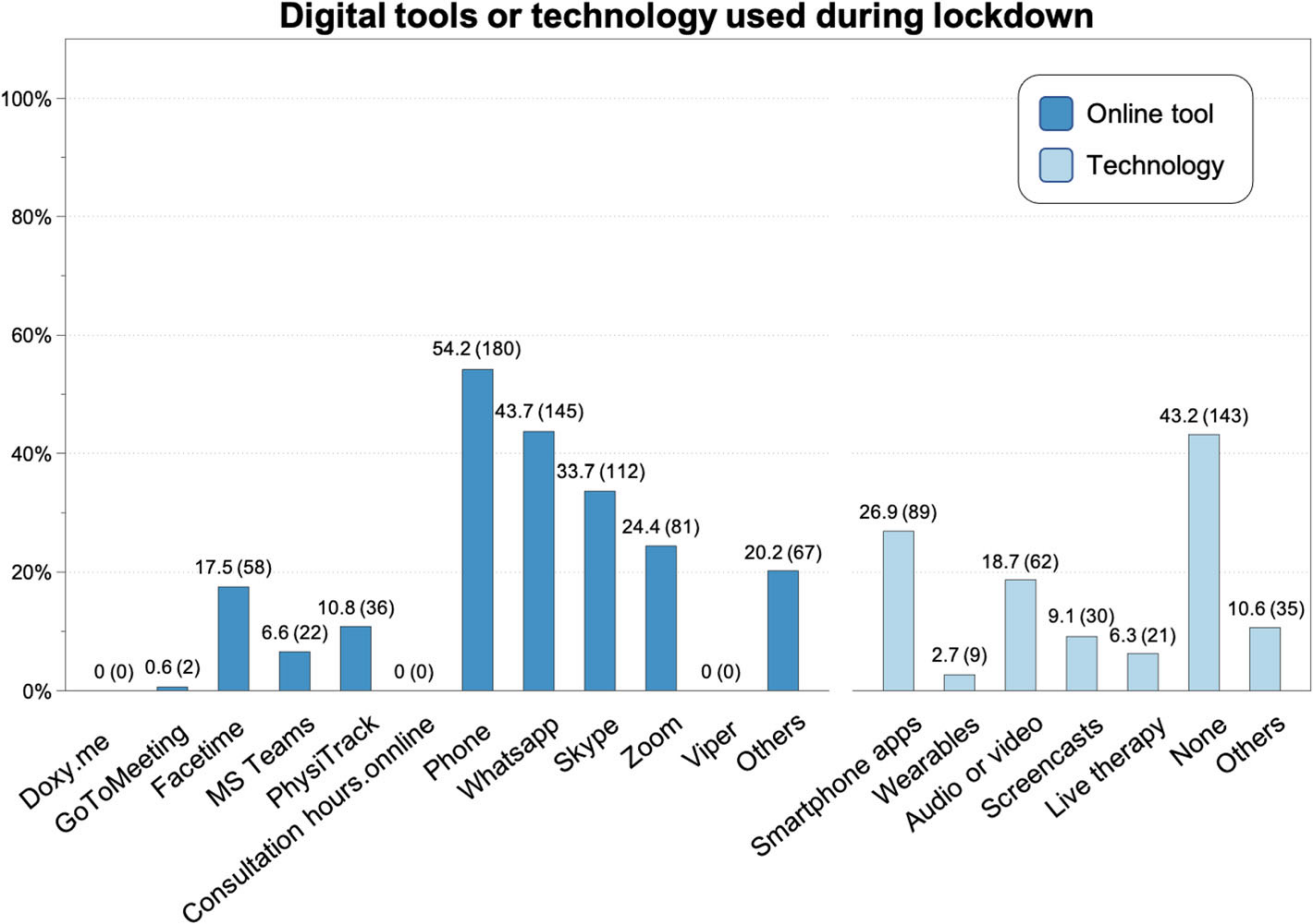
Digital tools or technology used during the lockdown by Swiss physiotherapists, percentage (absolute number)

The specific clinical applications of digital tools during the lockdown, and the tools most used by Swiss PTs, are depicted in Figs. 2 and 3, respectively.

The majority of those using the remote treatment modality stated they were interested in receiving more information or training regarding online therapy (*n* = 292, 88.2%). The areas of greatest formative interest are shown in detail in Table 3**.**

About two-thirds of those using remote physiotherapy adopted no additional data protection procedures (for example, a specific informed consent form) compared to the standards already in use. The most commonly used billing methods for this form of intervention were tariff item number 7301 (individual session of general physiotherapy, 30 min, approx. CHF 50) (*n* = 98, 29.6%), tariff item number 7340 (medical training instruction, 15 min, approx. CHF 25) (*n* = 55, 16.6%), and other forms (*n* = 37, 11.2%). However, a large proportion of physiotherapists offered their sessions without extra billing (*n* = 141, 42.6%).

#### Support - the PTs’ perceptions and evaluation of the use of digital remote physiotherapy

Almost half of the participants stated that compared to before the lockdown, communication and personal contact with patients could be maintained at about the same level through digital tools (*n* = 165, 49.8%). Two-thirds of the respondents (*n* = 222, 67.1%) were not confident (at all) that remote care could complement usual physiotherapy in the future, and 145 (43.8%) stated that they had no interest in continuing remote therapies after the COVID-19 pandemic. Twenty participants (6%) were (very) confident that digital remote therapy would be a worthwhile supplement to traditional physiotherapy, and 66 (19.9%) planned to continue with digital remote physiotherapy after the pandemic.

The reasons for the non-provision of digital remote therapies by the remaining sample are summarized in Table 3**,** “others” are described in Supplement material (S2).

## Discussion

This cross-sectional survey of 742 PTs practicing in CH revealed that the spring lockdown due to COVID-19 had a great impact on their working situation. Overall, the characteristics of the respondents are representative of the Swiss PT community. Swiss PTs reacted to the moratorium by adopting various low-cost and easily-accessible digital technologies to provide interventions to their patients, or at least to keep in communication with them. This proactive behavior certainly represents a professional commitment but does not necessarily mean that digital remote physiotherapy can be integrated easily into usual care practice in Switzerland.

The number of PTs providing digital remote physiotherapy during the lockdown increased from 4.9 to 44.6%, suggesting a positive context-driven adaptation and capability to respond to new demands in the healthcare system. This is remarkable in a profession historically determined by the “therapeutic touch” [21], a framework in clear contrast to the concept of digital remote physiotherapy.

Like many HPs throughout the world [22], Swiss PTs made use of low-cost and easily-accessible digital technologies, such as a mobile phone, smartphone applications (e.g., WhatsApp) and online meeting tools (e.g., Skype, Zoom). Treatments, patient-education, and follow-ups were provided remotely, even though the technologies utilized were not originally developed to support these healthcare activities. Prior to the lockdown, the adoption and use of digital devices and services had not been widespread in CH [23], even though comprehensive solutions were available (but not always sustainable) to complement and enrich traditional physiotherapy and benefit treatment outcomes [24–26].

For various reasons, despite PTs stating an interest in learning more about digital technology, the majority did not intend to work remotely in the future. A survey of Canadian PTs indicated that PTs largely have a positive attitude towards technology-based therapy (mobile or wearable) [27]. However, a US national survey of 500 clinicians completed in 2019 reported that only 50% of the PTs interviewed felt ‘very’ or ‘extremely comfortable’ about integrating mobile rehabilitation technologies into their clinical practices. In addition, only 30% of these PTs consider themselves to have adequate knowledge of the available technologies for their patients [28].

In order to reduce the regulatory and other professional barriers to this emerging mode of service delivery, which is urgently needed in times of a pandemic, the specific barriers and facilitators in the Swiss setting must be elaborated. Digital solutions and regulations, such as standards for the use of technologies, data security, and educational strategies must be developed to prepare for similar situations in the future. Digital remote therapy might also be conceived as a method of maintaining care in cases of shortened inpatient rehabilitation [29].

The implementation of healthcare innovations can be driven by patient needs or demands, scientific findings, ethical and legal requirements [30, 31]. In several countries, such as Australia [32], the USA [33], and Canada [34], the COVID-19 pandemic fostered the implementation of remote physiotherapy. Digital remote physiotherapy can reduce barriers to access for people living in rural areas, for those with mobility issues, for people with difficulty in taking time off work, and - of course - in the context of a pandemic, give access to those with limitations on their physical contact. Many countries realized the limitations of face-to-face consultations and supported HPs in finding creative solutions. However, HPs need to have access to devices and stable internet connections; they need to be educated in digital remote therapy; and to be reimbursed for it. Survey participants stated that they billed health insurance companies for digital remote therapy differently to what was specified by the Swiss Covid Law or offered pro bono services to stay in touch with patients. Appropriate reimbursement is important to not restricting patient access to appropriate digital care [1]. During the second lockdown in CH (Dec.20-Feb.21), new regulations recognized this problem and increased the number of accepted indications for digital remote therapy and adjusted the tariff position [35].

Evidence shows that eHealth literacy rises with social influence, performance expectancy, and education addressing misconceptions regarding inferiority and congruity with conventional treatment [36]. Australia integrated digital remote physiotherapy to entry-level education to promote PTs’ eHealth literacy [37].

Telemonitoring and telerehabilitation were strongly recommended for post-acute rehabilitation of people with COVID-19 [38] and other conditions [2]. However, digital remote therapy has several limitations and clearly cannot replace face-to-face therapy. Therefore, the time-point and style of use must be considered carefully: teletherapy might be inappropriate for a first physiotherapy session, since the recording of clinical history, clinical reasoning, and assessment cannot be performed so efficiently from a distance [39]. However, in exceptional situations, such as a pandemic, the PT should have the ability to decide on the value of teletherapy - even for a first session. Similarly, contextual factors, such as touch or clinical setting [8, 40], are lacking and therefore teletherapy should be enriched with tailored communication [39].

The needs from the perspective of Swiss patients must be explored. Literature describes various barriers (e.g., age, computer literacy [7]), but also cases of acceptance of and satisfaction with digital remote physiotherapy [41]. Patients have clearly stated that they consider remote therapy to be a suitable complement to face-to-face therapy in a blended approach, but not as a replacement of usual care [25].

The success of online consultation depends significantly on the digital competencies of both HPs and patients [42]. Virtual consultations and digital monitoring devices in physiotherapy create new methods to encourage treatment adherence and monitoring (including sharing of patient data with the PT). These may help to alter the perceptions of both patients and therapists of their treatment experience, as well as of their interaction during consultation.

Although digital remote therapy is not yet established in CH, stakeholders should be visionary and pave the way for change in this form of healthcare modality, e.g., by developing guiding principles and integrating digital remote therapy into entry-level physiotherapy curricula. Employers could enhance digitalization by providing organizational support and appropriate educational resources to strengthen HPs’ digital competencies, which could lead to more efficient workflow and improved patient care [43].

This was the first observational study on perceptions of the use of digital remote therapy by Swiss PTs. A strength of the survey was that it was supported by the Physiotherapy Institutes of the four Swiss Universities of Applied Sciences (UASs) and physioswiss, which underpins the high relevance and actuality of the topic. The survey was translated into the three main national languages of CH, thus avoiding a linguistic barrier. Findings may lead to several subsequent projects in research, teaching, and professional development.

There are, however, some limitations to this study. The large number of responses in the ‘free text’ answers indicate that the explanations of the answer options were either not appropriate or not sufficient, and that better explanatory examples could have been useful. More extensive pilot testing with a larger sample may have potentially helped to mitigate this, although, due to the pandemic, there was intense pressure to develop the survey at high speed to capture timely data. For transparency reasons, a supplement with additional information on the ‘free text’ answers is presented. Furthermore, the survey could have focused more on exploring the barriers and facilitators and deriving implications for the further development of digital remote therapy in the Swiss clinical context.

## Conclusion

During the first COVID-19 lockdown, Swiss PTs adopted various low-cost and easily accessible digital technologies to provide interventions to their patients. However, their attitudes towards the use and benefits of digital remote therapy were not found to be positive. Additionally, several barriers hampered further implementation of this modality. Specific education and training programs need to be provided for undergraduate and graduate physiotherapy, appropriate digital technologies should be introduced, and a correct reimbursement scheme should be developed.

## Supplementary Information

**Additional file 1.**

**Additional file 2.**

## Data Availability

The datasets used and/or analysed during the current study are available from the corresponding author on reasonable request.
