# Supplementary material for: Physiotherapists’ use and perceptions of digital remote physiotherapy during COVID-19 lockdown in Switzerland: an online cross-sectional survey
Source: Arch Physiother. 2021 Jul 7;11:18. doi: 10.1186/s40945-021-00112-3 (PMC8261812; doi:10.1186/s40945-021-00112-3)
Supplement: Supplementary file 1 — Additional file 1. [file 40945_2021_112_MOESM1_ESM.docx]

# Survey on the opportunities and limitations of remote, digital physiotherapy during and after the Corona pandemic

# Instructions on the questionnaire. Please read carefully before you start.

# What is this questionnaire about?

By means of a survey among physiotherapists in Switzerland, we would like to investigate the use and perceptions of digital, remote physiotherapy during and after the COVID-19 pandemic. The knowledge gained will contribute to being prepared for a comparable situation in the future.

- **Who can participate?** All physiotherapists working in Switzerland.
- **Privacy:** This survey is completely anonymous. This means that we do not collect any data about your identity; no conclusions about your person will be possible.
- **What happens with the data?** The answers are evaluated by researchers from the universities of applied sciences ZHAW, BFH, SUPSI, and HES-SO. The results will only be published in summary form and it will not be possible to draw conclusions about individual persons.
- **How long does it take to answer the questions?**

Answering the questions takes about 10-15 minutes.

If you have any questions or suggestions regarding the survey, please contact us via e-mail:

Heiner Baur, [heiner.baur@bfh.ch](mailto:heiner.baur@bfh.ch), BFH (German)

Veronika Schoeb, [veronika.schoeb@hesav.ch](mailto:veronika.schoeb@hesav.ch), HES-SO (French)

Marco Barbero, [marco.barbero@supsi.ch](mailto:marco.barbero@supsi.ch), SUPSI (Italian)
Anne-Kathrin Rausch, [anne-kathrin.rauschosthoff@zhaw.ch](mailto:anne-kathrin.rauschosthoff@zhaw.ch), ZHAW (German)

**I have read the information about this survey and agree to participate:**

- Yes
- No

|  | **Category of**  **questions** | **Variable** | **Question** | **Values** |
| --- | --- | --- | --- | --- |
| 1 | Demography | Gender | Are you | male, female, other, I don’t want to specify |
| 2 |  | Age | What year were you born in? |  |
| 3 |  | Work experience | In which year did you obtain your basic  physiotherapy license? |  |
| 4 | Attitude towards technology | Use of digital tools | Which digital tools do you use for personal purposes? | Smartphone apps (Whatsapp, Instagram, Twitter, Youtube, etc.)  Online Meeting tools (Zoom, Skype, MS Teams, etc.)  Smart Watches  Other wearables |
| 5 |  |  | Which digital tools do you use for professional purposes (e.g. patient contact, exchange with colleagues, professional training)? | Smartphone apps (Whatsapp, Instagram, Twitter, Youtube, etc.)  Online Meeting tools (Zoom, Skype, MS Teams, etc.)  Smart Watches  Other wearables |
| 6 |  | Frequency of use of digital tools | How often do you use digital tools for personal purposes? | Never  1-2 times per months  Once per week  3-5 times per week  Every day |
| 7 |  |  | How often do you use digital tools for professional purposes? | Never  1-2 times per months  Once per week  3-5 times per week  Every day |
| 8 | Activity as PT | Patient contact | Do you have contact with patients during your work as physiotherapist? | Yes 🡪 Q9  No 🡪 STOP |
| 9 |  | If Q8 YES | What kind of patients do you mainly see working as physiotherapist? | - The outpatient sector (e.g. practice) - The inpatient sector (e.g. hospital, rehabilitation clinic) - both |
| 10 |  | Workload before_1 | Which of the following activities were part of your duties BEFORE THE LOCKDOWN, and what percentage of the job was allocated to them? | - Patient contact - Administration (e.g. personnel, payroll) - Teaching - Research - Other |
| 11 |  | Workload before_2 | How many effective working hours per week did you work BEFORE THE LOCKDOWN (start 16.3.2020)? |  |
| 12 | Corona | Workload during_1 | Which of the following activities were part of your duties DURING THE LOCKDOWN, and what percentage of the job was allocated to them? | - Patient contact - Administration (e.g. personnel, payroll) - Teaching - Research - Other |
| 13 |  | Workload during_2 | How many effective working hours per week did you work DURING the lockdown (16.3.2020 to 27.4.2020)? |  |
| 14 |  | Change of function | Have you been assigned to another function at your workplace DURING the lockdown (16.3. to 27.4.2020)? | - Yes, in the field of physiotherapy 🡪 Q15 - Yes, outside the field of physiotherapy 🡪 Q16 - No |
| 15 |  | If within field PT | What new/other function did you have during the lockdown? | - Respiratory therapy/care of Covid Pat in intensive care - Change to another Physio- specialized field - Another, namely (limit 100 characters) |
| 16 |  | If within field non-PT | What new/other function did you have during the lockdown? | - Triage - Nursing of non-covid patients - contact tracing - Administration (e.g. billing) - Organization of procedures (e.g. patient transport) - Another, namely... (limit 100 characters) |
| 17 | Technical Tools |  | Did you already offer video-tele/online- therapy  BEFORE the lockdown (start 16.3.2020)? | Yes  No |
| 18 |  |  | Did you offer video tele/online therapy DURING the  lockdown (16.3.-27.4.2020)? | Yes 🡪 Q19 - 29  No 🡪 Q30 |
| 19 |  | If Q18 yes | In which part of patient management/phase of therapy did you use digital technology? | - History taking - Examination and evaluation - Define a diagnosis - Identify a prognosis - Provide the treatment - Patient education - Follow up the clinical course - Improve the treatment adherence (e.g. sending reminder) - Therapy monitoring (e.g. outcome assessment) |
| 20 |  | If Q18 yes | In which setting did you offer tele/online therapy? | Individual therapy  Group therapy |
| 21 |  | If Q18 yes | To which patients did you offer of tele/online therapy? | - Patients who belong to the COVID-19-risk group - Patients with COVID-19 - Indication Musculoskeletal - Indication geriatrics - Indication Internal organs and vessels - Indication neuromotor and sensory - Indication Pediatrics - Other... (limit 100 characters) |
| 22 |  | If Q18 yes | Which tool did you use to perform tele/online | - Doxy.me |
|  |  |  | therapy with your patients? | - Go to meeting |
|  |  |  |  | - Facetime |
|  |  |  |  | - Microsoft teams |
|  |  |  |  | - Physitrack |
|  |  |  |  | - Consultation hours.online |
|  |  |  |  | - Phone - Viper WhatsApp - Skype - Zoom - Other things that... (limit 100 characters) |
| 23 |  | IfQ18 Yes | Did you use other digital technology beside tele/online therapy with you patients? | Smartphone apps (e.g. digital exercise prescription for home training, etc.).  Wearables, smart objects (e.g. accelerometry, force sensores, fitbit etc.)  Passing audio (podcast) or video information (e.g. youtube)  Own screencasts  Own live therapy for group training  None  Others, namely... |
| 24 |  | IfQ18 Yes | What actions have you taken regarding data protection? | Consent form  None  Others, namely... |
| 25 |  | If Q18 yes | How do you charge for Video-Tele/Online Therapy? | - Not at all - 7340 (22 taxpoints) - 7301 (48 taxpoints) - Different,.. |
| 26 |  | If Q18 yes | Will you keep offering video tele/online therapy after Corona? | Yes  No  Uncertain |
| 27 | Support | If Q18 yes | Compared to the times before the lockdown, how do you judge the communication/personal contact with your patients DURING the lockdown? | Much better, better, neutral, worse, much worse |
| 28 |  | If Q18 yes | Compared to the times before the lockdown, how do you judge the quality of the therapeutic interventions (e.g. instruction and counselling via video) DURING the lockdown? | Much better, better, neutral, worse, much worse |
| 29 |  | If Q18 yes | How confident are you that tele/online therapy can be a worthwhile complement to the usualphysiotherapy? | Very confident, confident, neutral, not confident, not confident at all |
| 30 |  | If Q18 NO | Why did you not offer tele/online therapy? | - I was able to provide my patients with sufficient care in another way. - I cannot observe the patient adequately. - I miss the tactile control/possibility of manual support - Online/teletherapy is not adequately reimbursed. - The technical possibilities are unknown to me or my patients. - The necessary infrastructure is missing for me/my patient. - The other reason is.... |
| 26 |  | Support | Would you like to have more information/a  training offer regarding Video-Tele/Online Therapy? | Yes 🡪 Q27  No |
| 27 |  | If Q26 yes | What form of support would you find useful? | - Knowledge about infrastructure - Knowledge about applications (Apps) - Knowledge about law and data protection - Knowledge about settlement with cost units - Knowledge of federal and cantonal ordinances - Knowledge about the needs of patients - Knowledge of patient requirements - Knowledge about effectiveness - Knowledge of communication methods - Knowledge of the examination and treatment process - Knowledge of suitable methods - Knowledge of other (please specify) |

# Would you like to receive the results of this survey?

- Yes
- No
- If yes, please enter your e-mail address. This will be treated confidentially and will not be passed on to third parties:

# If we conduct another survey/interviews on the subject, would you allow us to contact you directly by email?

- Yes
- No
- If yes, please enter your e-mail address. This will be treated confidentially and will not be passed on to third parties:

# We thank you for your cooperation. Stay healthy!

# Kind regards, on behalf of the project team
