# Supplementary material for: Physiotherapists’ use and perceptions of digital remote physiotherapy during COVID-19 lockdown in Switzerland: an online cross-sectional survey
Source: Arch Physiother. 2021 Jul 7;11:18. doi: 10.1186/s40945-021-00112-3 (PMC8261812; doi:10.1186/s40945-021-00112-3)
Supplement: Supplementary file 2 — Additional file 2. [file 40945_2021_112_MOESM2_ESM.docx]

**Answers to «other» option**

| **Attitude towards technology** | | **n** | **%** |
| --- | --- | --- | --- |
| Which digital tools do you use for personal purposes? (n=57) | PC, Laptop, Tablet | 29 | 51 |
|  | E-Mail | 12 | 21 |
|  | SMS/phone | 4 | 7 |
|  | Online shopping/booking, google | 3 | 5 |
|  | None | 3 | 5 |
|  | Online Meeting Tools* | 3 | 5 |
|  | Smartphone Apps* | 2 | 4 |
|  | Smart Watch* | 1 | 2 |
| Which digital tools do you use for professional purposes (e.g. patient contact, exchange with colleagues, professional training)? (n=174) | E-Mail | 61 | 3  5 |
|  | PC, Laptop, Tablet | 45 | 26 |
|  | SMS/phone | 23 | 13 |
|  | Applications for medical use (e.g. Physitrack, Medicosearch) | 15 | 9 |
|  | Clinic system | 9 | 5 |
|  | Smartphone Apps* | 7 | 4 |
|  | None | 5 | 3 |
|  | Online Meeting Tools* | 4 | 2 |
|  | Data storage, data transfer | 3 | 2 |
|  | Homepage | 2 | 1 |
| **PT activity during Corona** | | **n** | **%** |
| Which of the following activities were part of your duties DURING THE LOCKDOWN, and what percentage of the job was allocated to them? (n=245) | Organisation/procedures in clinic | 42 | 17 |
|  | Patient contact* | 38 | 16 |
|  | Administration (e.g. personnel, payroll)* | 31 | 13 |
|  | Therapeutic activities (e.g. home exercise program) | 30 | 12 |
|  | Renovations/cleaning | 26 | 11 |
|  | Temporary help (e.g. nursing) | 20 | 8 |
|  | Nothing/short-time work/child care | 17 | 7 |
|  | Further education, projects | 16 | 7 |
|  | Other jobs (e.g. IT service, dance teacher) | 9 | 4 |
|  | Teaching* | 6 | 2 |
|  | Research* | 6 | 2 |
|  | Professional politics, physio association | 4 | 2 |
| What new/other function did you have during the lockdown WITHIN the field of physiotherapy? (n=40) | Administration, organisation, home exercise programs | 17 | 43 |
|  | Project concepts | 17 | 43 |
|  | Change to another Physio- specialty* | 3 | 8 |
|  | Respiratory therapy of COVID-19 patients in intensive care* | 1 | 3 |
|  | Professional association | 1 | 3 |
|  | Home visits | 1 | 3 |
| What new/other function did you have during the lockdown OUTSIDE field of physiotherapy? (n= 28) | Other activities related to COVID-19 but not nursing | 7 | 25 |
|  | Temporary help | 6 | 4 |
|  | Cleaning | 5 | 18 |
|  | Organization of procedures* | 3 | 11 |
|  | IT | 3 | 11 |
|  | Nursing of non COVID-19 patients* | 2 | 7 |
|  | Administration* | 1 | 4 |
|  | Others (courier service, child care) | 1 | 4 |
| Why did you not offer tele/online therapy? (n=72) | Not allowed by superior/head of institution | 17 | 24 |
|  | Not possible with target group | 12 | 17 |
|  | Setting not appropriate | 12 | 117 |
|  | Several of the mentioned reasons | 8 | 11 |
|  | I miss the tactile control/possibility of manual support* | 6 | 8 |
|  | Not wanted by patients | 4 | 6 |
|  | Online/teletherapy is not adequately reimbursed* | 4 | 6 |
|  | I cannot observe the patient adequately* | 4 | 6 |
|  | I was able to provide my patients with sufficient care in another way* | 2 | 3 |
|  | The technical possibilities are unknown to me/my patients* | 2 | 3 |
|  | The necessary infrastructure is missing for me/my patient* | 1 | 1 |
| To which patients did you offer of tele/online therapy? (n=35) | All interested patients or those who did not want other treatment | 13 | 37 |
|  | Special diagnosis | 7 | 20 |
|  | Indication Gynaecological | 4 | 11 |
|  | Pain, psychosomatic | 3 | 9 |
|  | Internal organs and vessels* | 3 | 9 |
|  | Group exercise therapy | 2 | 6 |
|  | Indication Musculoskeletal* | 2 | 6 |
|  | Patients who belong to the COVID-19-risk* | 1 | 3 |
| **Technical tools** | | **n** | **%** |
| Which tool did you use to perform tele/online  therapy with your patients? (n=69) | E-mails | 22 | 32 |
|  | Other apps, mentioned once | 11 | 16 |
|  | Medicosearch | 9 | 13 |
|  | Webex | 6 | 9 |
|  | Jitsi meet | 4 | 6 |
|  | Google | 4 | 6 |
|  | Physioapps e.g. mayway, physiotec | 3 | 4 |
|  | Starleaf | 2 | 3 |
|  | HIN | 2 | 3 |
|  | Physitrack* | 2 | 3 |
|  | Postal mail | 1 | 1 |
|  | Facetime* | 1 | 1 |
|  | Skype* | 1 | 1 |
|  | Phone* | 1 | 1 |
| Did you use other digital technology beside tele/online therapy with you patients? (n=32) | E-mails | 11 | 34 |
|  | Others (Instagram, online programs of services) | 7 | 22 |
|  | Smartphone apps* | 4 | 13 |
|  | Postal mail of exercise programs | 2 | 6 |
|  | Live online treatment/instructions | 2 | 6 |
|  | Own screencasts* | 2 | 6 |
|  | None* | 2 | 6 |
|  | Internal app | 1 | 3 |
|  | Sharing audio (podcast) or video information* | 1 | 3 |
| What actions have you taken regarding data protection? (n=55) | Verbal agreement | 31 | 56 |
|  | Secure tools used | 13 | 24 |
|  | Informed consent forms integrated in tools | 4 | 7 |
|  | Not needed | 3 | 5 |
|  | Institution organised informed consent | 3 | 5 |
|  | Consent via e-mail* | 1 | 2 |
| How do you charge for Video-Tele/Online Therapy? (n=38) | 7301 (48 taxpoints)* | 12 | 32 |
|  | 7311 | 9 | 24 |
|  | Not at all* | 5 | 13 |
|  | Do not know yet | 5 | 13 |
|  | Self-payer | 3 | 8 |
|  | 7340 (22 taxpoints)* | 3 | 8 |
|  | Financed by a project | 1 | 3 |
| **Support** | |  |  |
| What form of support would you find useful? (n=5) | Knowledge of settlement with cost units* (n=2) | 2 | 40 |
|  | Knowledge of work life balance | 1 | 20 |
|  | Knowledge of applications (Apps)* | 1 | 20 |
|  | Knowledge of behaviour change regarding physical activity | 1 | 20 |

*Categories, which were already part of the questionnaire
